# Achieving change readiness for health service innovations

**DOI:** 10.1111/nuf.12713

**Published:** 2022-02-19

**Authors:** Reema Harrison, Ashfaq Chauhan, Huong Le‐Dao, Amirali Minbashian, Ramesh Walpola, Sarah Fischer, Gavin Schwarz

**Affiliations:** ^1^ Centre of Health Systems and Safety Research, Australian Institute of Health Innovation Macquarie University Sydney New South Wales Australia; ^2^ School of Population Health UNSW Sydney Sydney New South Wales Australia; ^3^ School of Management and Governance, UNSW Business School UNSW Sydney Sydney New South Wales Australia; ^4^ Clinical Excellence Commission, NSW Health Sydney New South Wales Australia

**Keywords:** change readiness, innovation, workforce

## Abstract

Continual innovation to address emerging population needs necessitates health service ongoing redesign and transformation worldwide. Recent examples include service transformations in response to Covid‐19, many of which were led and managed by nurses. Ensuring change readiness is central to delivering these transformative changes yet has been identified as a central challenge impacting nurse leaders and managers. Recent evidence indicates that affective commitment to change among healthcare staff may be an important contributor to gaining support for change implementation but understudied in healthcare. A cross‐sectional survey study was used to examine the association between affective commitment to change and change readiness among 30 healthcare staff across four projects in one state‐wide health system in Australia. Our findings indicate that affective commitment to change; healthcare worker's emotional and personal perception of the value of the proposed change is independently associated with individual and collective change readiness. Given that achieving change readiness is a central goal of change management strategies, this pilot work provides valuable insight to inform the change management practices of nurse leaders and managers.

## 1 INTRODUCTION

Continual innovation to address emerging population needs necessitates health service ongoing redesign and transformation worldwide. Recent examples include service transformations in response to Covid‐19. The pandemic has catalyzed change with regard to how healthcare is delivered by promoting rapid acceleration in the use of virtual models of care.^1^ As work processes, systems, and models of care shift in response to health system needs, so too must individual and collective behaviors of the workforce and its consumers.^2^, ^3^ Ensuring effective change management processes occur is therefore central to transformative changes that deliver their intended outcomes and are sustained. Yet change management is notoriously challenging in healthcare contexts. Nurse leaders and managers identify achieving change readiness as a key challenge that requires evidence and implementation support.^4^

Achieving “change readiness” among healthcare staff is identified as an important precursor to whether staff accept and adopt a change initiative. Change readiness can be defined as the extent to which staff perceive that a given change is needed (their commitment to the change) and that they have the required capability and support to work in a new way (change self‐efficacy).^5^, ^6^ Readiness for change has been conceptualized at the individual level, workgroup, and organizational levels, with associations between individual and collective change readiness theorized.^5^ Commitment to change arises for three reasons: (a) because there is a requirement to support the change due to recognition of cost associated with failure to do so (continuance commitment), (b) because there is a sense of obligation to support the change (normative commitment), and (c) because an individual wants or desires to support the change due to the benefits associated with it (affective commitment).^7^, ^8^ There are identified conceptual gaps around the role of affect that we seek to explore and address through this study.^5^

Recent evidence indicates that affective commitment to change is an important contributor to achieving change readiness but understudied in relation to healthcare projects.^9^, ^10^, ^11^ A systematic review of 38 studies of healthcare change projects highlights that management approaches are often led by nursing staff but also rarely focus on the influence of affective commitment to change.^12^ Our analysis sought to address the evidence gap regarding the association between affective commitment to change and change readiness in healthcare by exploring change readiness among clinical and nonclinical staff directly involved in one of four transformational change projects in a state‐wide health system in Australia. The study aimed to (a) establish the level of individual and collective change readiness among healthcare staff, and (b) establish whether there is an association between affective commitment to change and change self‐efficacy and change readiness. We hypothesized that affective commitment to change would be positively associated with change readiness.

## 2 METHOD

### 2.1 Ethical approval

This study was approved by Western New South Wales Human Research Ethics Committee (approval number 2020/ETH01247).

### 2.2 Design

Cross‐sectional survey.

### 2.3 Survey instrument

A survey tool was developed by the research team consisting of five elements. The responses were measured on a 7‐point Likert scale (from 1—*strongly disagree* to 7—*strongly agree*). The four validated elements were: (i) individual change readiness scale (four items), (ii) collective change readiness scale (four items), (iii) change self‐efficacy scale (four items), and (iv) affective commitment to change scale (three items).^7^, ^13^ A fifth element contained purposively developed items to assess participants' understanding and awareness of the change being proposed.

### 2.4 Setting

The study was conducted in partnership between academic researchers, health agencies, and two local health districts (LHDs) in NSW, Australia. Two LHDs in Australia participated in this study, one servicing a metropolitan region and one servicing a rural/remote region. Within each district, the project partners identified two transformational change projects that were at a suitable stage to address our aims. This meant that projects were of a similar scale, at a similar point in their planning, but implementation had not yet commenced. The four participating projects were: virtual pharmacy services, in‐home monitoring services, emergency department expansion, and an outpatient administration redesign.

### 2.5 Recruitment and procedure

Employees in any clinical or nonclinical role who were directly affected by or involved in the change/s proposed in each project were eligible to participate: a total participant pool of 60 participants was available who met the eligibility criteria in the participating projects. A minimum sample size of 15 participants per site was sought to enable us to detect significant changes in individual change readiness between sites.^14^ Participants were recruited via a study invitation email with an embedded survey link. The email was distributed by the research team to eligible potential participants at each site through the local study partners. Written consent was obtained from individuals before their participation.

### 2.6 Analysis

Descriptive and inferential statistics for sample characteristics and the levels of change readiness, change commitment, and change self‐efficacy were explored using SPSS (IBM Statistics, Version 25). Pearson correlation coefficients were calculated to examine the relationships between readiness for change (individual and collective), change self‐efficacy, and affective commitment to change. Multiple regression analysis using ordinary least square estimation was conducted to examine the independent effects of the two antecedents (affective commitment to change and change self‐efficacy) on individual change readiness. Specifically, we conducted a simultaneous regression in which change readiness was entered as the outcome variable and change self‐efficacy and affective commitment to change were entered as predictors.

## 3 RESULTS

Thirty participants of the 47 invited (64% response rate) completed the survey. Table 1 provides a breakdown of participant characteristics by site and overall.

**Table 1:** Demographic information

Characteristics	LHD1	LHD2	Total
Gender			
Male	0	2	2 (6.7%)
Female	14	14	28 (93.3%)
Total	14	16	30 (100%)
Current role in the project			
Clinician	8	13	21 (70%)
Nonclinician	1	0	1 (3.3%)
Change sponsor	2	0	2 (6.7%)
Manager	1	3	4 (13.3%)
Other	2	0	2 (6.7%)
Length of employment with the health district			
<2 years	2	3	5 (16.7%)
2−5 years	3	1	4 (13.3%)
6−10 years	2	2	4 (13.3%)
>10 years	7	10	17 (56.7%)
Type of employment			
Perm part‐time	1	5	6 (20%)
Perm full‐time	12	9	21 (70%)
Fixed term	1	2	3 (10%)

Participants from both sites scored highly on individual and group readiness for change (Table 2). Readiness for change (individual and collective), change self‐efficacy, and affective commitment to change were moderately to strongly correlated in a positive direction. Higher affective commitment to change was associated with higher levels of self‐efficacy (*r* = .46, *p* < . 01) and individual readiness for change (*r* = .75, *p* < .001). The correlation between self‐efficacy and individual change readiness was moderate in size (*r* = .32) but not statistically significant (*p* = .08). Finally, collective readiness for change was positively and significantly associated with higher levels of change self‐efficacy, affective commitment to change, and individual change readiness (change self‐efficacy: *r* = .43, *p* = .02; affective commitment: *r* = .69, *p* < .001; individual change readiness: *r* = .39, *p* = .04).

**Table 2:** Descriptive statistics

Outcome variable	LHD 1 (*M*/SD)	LHD 2 (*M*/SD)	Total
Individual change readiness	6.47 (0.93)	5.89 (0.91)	6.15 (0.95)
Collective change readiness	6.09 (0.58)	5.73 (1.17)	5.87 (0.93)
Change self‐efficacy	5.55 (0.89)	5.48 (0.92)	5.40 (0.95)
Affective commitment to change	6.55 (0.64)	6.00 (0.92)	6.21 (0.86)

Regression analyses (Table 3) demonstrated that the effect of affective commitment to change on change readiness continued to remain strong after controlling for change self‐efficacy (*β* = .76, *p* < .001), whereas the effect of change self‐efficacy became negligible after controlling for affective commitment to change (*β* = −.03, *p* = .82).

**Table 3:** Regression analysis: independent effects of affective commitment to change and change self‐efficacy on individual change readiness

	*b*	SE	*t*	*p*	*β*
Intercept	0.836	1.008	0.830	.414	
Affective commitment to change	0.877	0.165	5.302	.000	.763
Change self‐efficacy	−0.036	0.158	−0.225	.823	−.032

*Note*: *N* = 30.

Abbreviations: *b*, unstandardized regression coefficient; *β*, standardized regression coefficient; SE, standard error.

Individual items in the fifth survey component (Table 4) indicated that participants were aware of the change and its success measures. The scores also indicated that most participants understood the criteria for success, felt they had leaders that could drive the change, and could get the support they needed. Although most felt that they had sufficient resources for change, this item had a slightly lower mean score overall (4.8/7) and wider variation between respondents.

**Table 4:** Perceptions of leadership, support, and success measures

Item (*n* = 30)	Disagree (*n*)	Neutral (*n*)	Agree (*n*)	Mean (SD)
I understand the criteria for success and how the change will be measured	3	3	24	5.2 (1.32)
We have the right leaders in key roles to drive the change	2	5	22	5.6 (1.35)
The resources assigned to the project are adequate to successfully deliver the change	4	7	19	4.8 (1.40)
I can get support when I have problems and questions	1	7	22	5.8 (1.30)

## 4 DISCUSSION

Our findings indicate that affective commitment to change, healthcare worker's emotional and personal perception of the value of the proposed change, is independently associated with individual and collective change readiness. This builds upon previous conceptual analysis regarding change readiness and the role of affect within this. Given that achieving change readiness is a central goal of change management strategies, this pilot work provides valuable insight to inform the change management practices of nurse leaders and managers. A larger scale replication of this analysis is warranted to explore these findings across health systems and a wider range of change projects.

Many factors contribute to an employee's affective commitment to change. Factors that contribute to an employee's affective commitment to change include employee's interpersonal workplace relationships with managers and with colleagues, change frequency (more frequent change reduces commitment to change), organizational communication about change, and employee's participation or engagement in decisions about the change.^15^ Nurse leaders and managers who wish to enhance affective commitment to change must therefore consider how they can influence these factors to promote change readiness. Techniques might include investing in enhancing interpersonal relationships with and between team members, managing the frequency and volume of change that is conveyed to staff by grouping multiple change efforts into single multimodel projects, and moderating organizational communications about changes ahead to manage the way that this information is received by staff, with opportunities to gather localized feedback and input.

Wider research literature indicates that employee engagement in decisions about the change process has been linked with individuals feeling more positive emotions toward change proposals and a greater understanding of the purpose of change proposals and the possible gains to be made. These reactions, in turn, are associated with a greater likelihood of employees making the behavioral changes required for changes to be adopted and sustained.^16^ When employees engage in decision‐making about changes and how change occurs, it also promotes interpersonal trust, attachment to their organization, and their sense of competence to achieve the changes needed.^17^ Employee engagement in decision‐making about changes may also, therefore, be a factor to consider in attempts to promote affective commitment to change.

Our findings must be considered in light of the limitations of the research. As a pilot project, the results need to be treated cautiously due to the small sample size and cross‐sectional nature of this study. Change readiness is not static and it is important to consider in future analysis how the environmental factors (e.g., the extent to which a health service environment has a culture, i.e., conducive to change, the level of resourcing and support for changes to be made successfully and leadership) influence whether change readiness is achieved and sustained. The sample was also made up largely of females and of clinicians who consented to participate, which may have also influenced the resulting data.

## 5 CONCLUSION

Our analysis provides novel insights into how nurse leaders and managers, along with other health system staff, might seek to address the challenge of bringing about change in health services. Our findings suggest a focus on value‐based approaches to achieve staff investment in change proposals may improve change readiness. These findings offer opportunities for nursing research and practice in approaches to lead and manage change by creating a basis for strong staff engagement.

## CONFLICT OF INTERESTS

The authors declare that there are no conflict of interests.

## Data Availability

Data can be made available upon request subject to ethical approval requirements being met.
